# Supplementary material for: Citizen Social Lab: A digital platform for human behavior experimentation within a citizen science framework
Source: PLoS One. 2018 Dec 6;13(12):e0207219. doi: 10.1371/journal.pone.0207219 (PMC6283465; doi:10.1371/journal.pone.0207219)
Supplement: S1 Fig — Screenshots of the initial screen of three experiments (a) Mr. Banks, (b) Dr. Brain and (c) The Climate Game. In this screen we introduce a character and a narrative to attract the attention of the public and to motivate them to participate. (PDF) [file pone.0207219.s001.pdf]

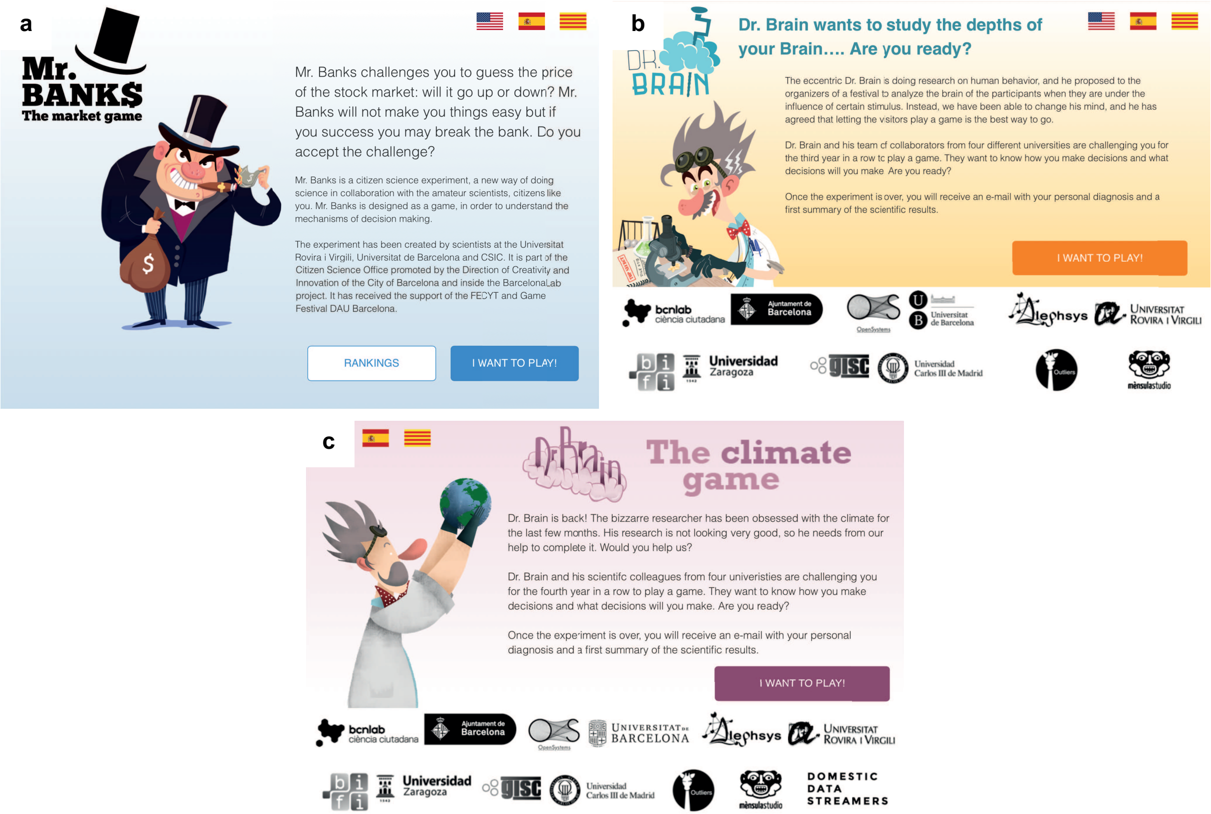

Fig S1: Home screen of Mr.Banks, Dr.Brain and The Climate Game. Screenshots of the initial screen of three experiments (a) Mr. Banks, (b) Dr. Brain and (c) The Climate Game. In this screen we introduce a character and a narrative to attract the attention of the public and to motivate them to participate. Images of the character created by Mensula Studio are licensed under CC BY 4.0.
