# Supplementary material for: Citizen Social Lab: A digital platform for human behavior experimentation within a citizen science framework
Source: PLoS One. 2018 Dec 6;13(12):e0207219. doi: 10.1371/journal.pone.0207219 (PMC6283465; doi:10.1371/journal.pone.0207219)
Supplement: S2 Fig — Screenshots of the main user interface of three experiments (a) Mr. Banks, (b) Dr. Brain and (c) The Climate Game where the participants respond to the dilemmas. (PDF) [file pone.0207219.s002.pdf]

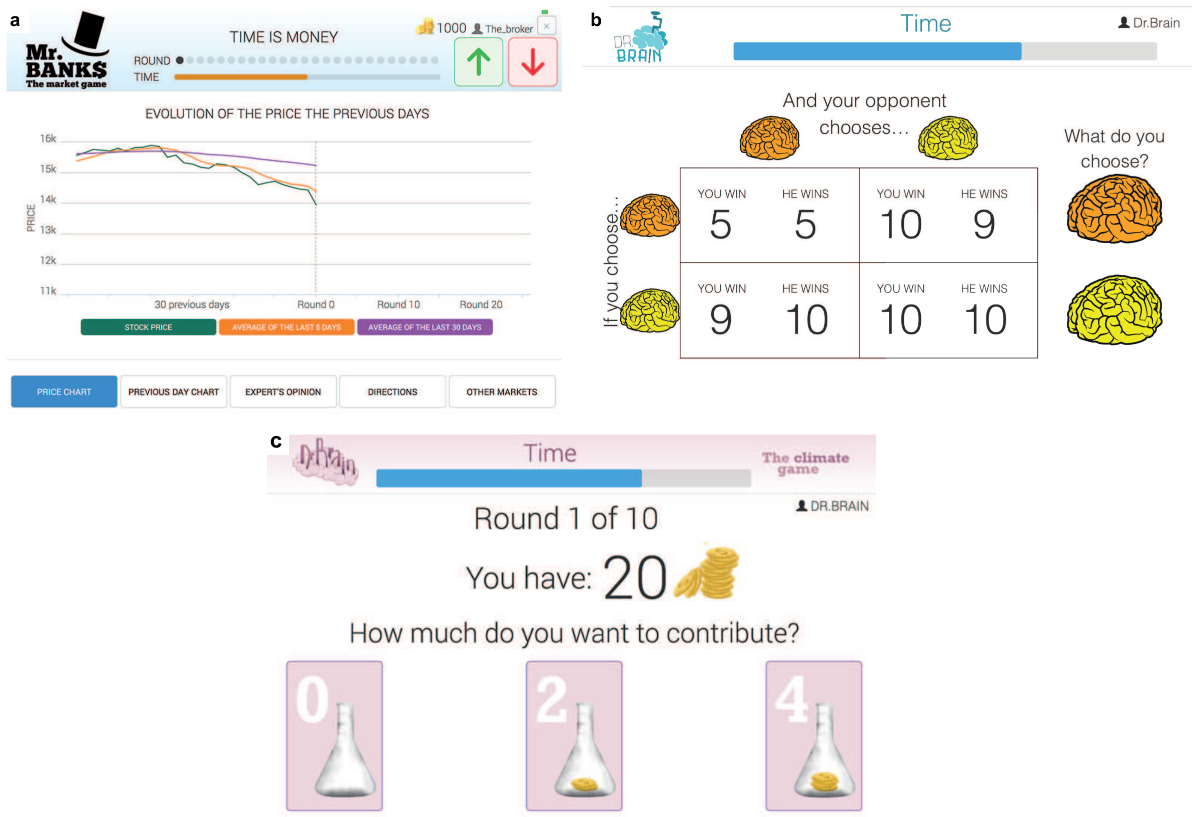

Fig S2: Decision-making interface of Mr.Banks, Dr.Brain and The Climate Game. Screenshots of the main user interface of three experiments (a) Mr. Banks, (b) Dr. Brain and (c) The Climate Game where the participants respond to the dilemmas. Images of the character created by Mensula Studio are licensed under CC BY 4.0.
