# Supplementary material for: Citizen Social Lab: A digital platform for human behavior experimentation within a citizen science framework
Source: PLoS One. 2018 Dec 6;13(12):e0207219. doi: 10.1371/journal.pone.0207219 (PMC6283465; doi:10.1371/journal.pone.0207219)
Supplement: S3 Fig — Screenshots of the tutorial shown before The Climate Game experiment where the participants learn the game mechanics and familiarize with the user interface. (PDF) [file pone.0207219.s003.pdf]

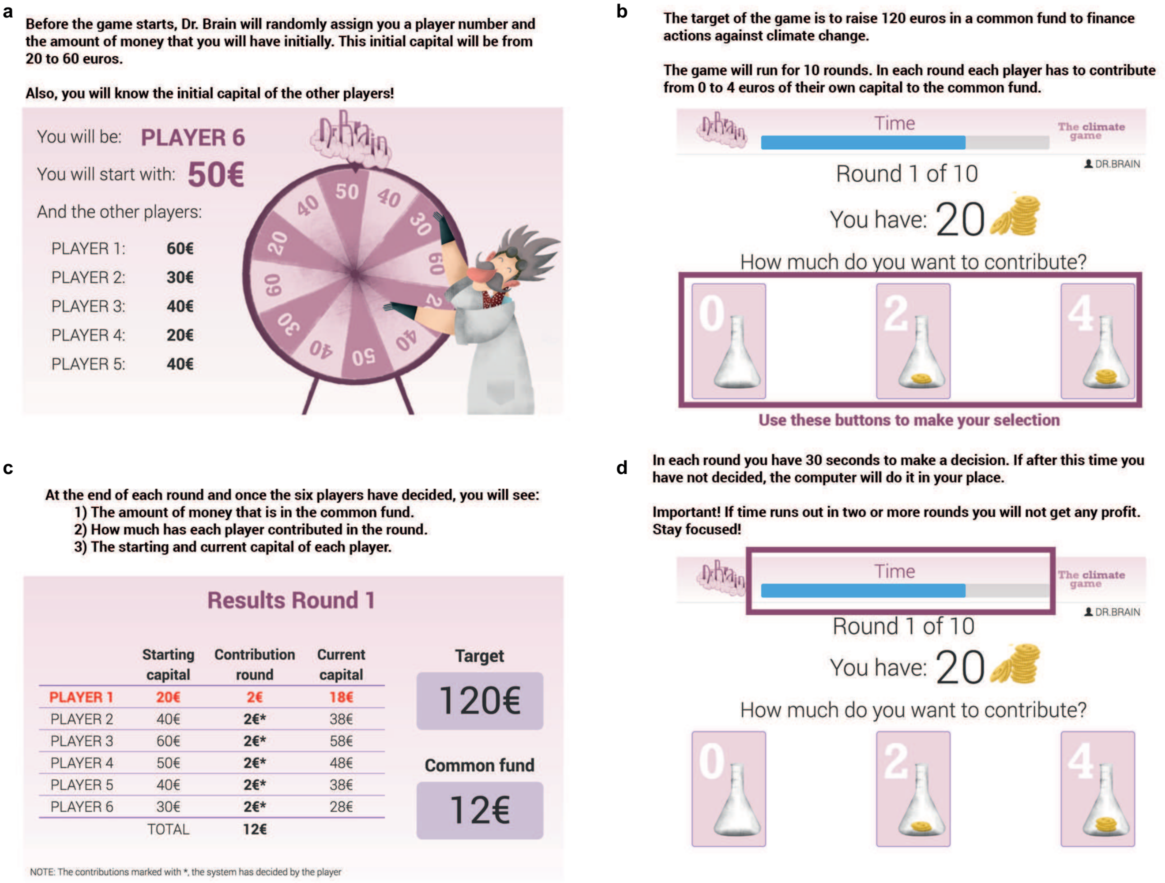

Fig S3: Tutorial interface of The Climate Game. Screenshots of the tutorial shown before The Climate Game experiment where the participants learn the game mechanics and familiarize with the user interface. Images of the character created by Mensula Studio are licensed under CC BY 4.0.
