# Supplementary material for: Citizen Social Lab: A digital platform for human behavior experimentation within a citizen science framework
Source: PLoS One. 2018 Dec 6;13(12):e0207219. doi: 10.1371/journal.pone.0207219 (PMC6283465; doi:10.1371/journal.pone.0207219)
Supplement: S1 Table — Biases with respect to the market (Participant/Market). (PDF) [file pone.0207219.s004.pdf]

Table S1: Market imitation. Biases with respect to the market (Participant/Market)

Experiment	Up/Up	Up/Down	Down/Up	Down/Down
DAU	0.71	0.29	0.47	0.53
CAPS	0.71	0.29	0.49	0.51
Sonar+D	0.78⋆	0.22	0.46	0.54

⋆ There are significant differences (-2.53 SD) between DAU and Sonar+D experiments (Binomial process differences test).
