# Supplementary material for: Citizen Social Lab: A digital platform for human behavior experimentation within a citizen science framework
Source: PLoS One. 2018 Dec 6;13(12):e0207219. doi: 10.1371/journal.pone.0207219 (PMC6283465; doi:10.1371/journal.pone.0207219)
Supplement: S2 Table — Decision conditioned to performance (Strategy/Decision). (PDF) [file pone.0207219.s005.pdf]

Table S2: Win-Stay Lose-Shift strategy. Decision conditioned to performance (Strategy/Decision)

Experiment	Win/Stay	Win/Shift	Lose/Stay	Lose/Shift
DAU	0.68	0.32	0.42	0.58
CAPS	0.71	0.29	0.48	0.52*
Sonar+D	0.72	0.28	0.40	0.60

* There are significant differences (2.35 SD) between DAU and CAPS experiments (Binomial process differences test).
