# Supplementary material for: Citizen Social Lab: A digital platform for human behavior experimentation within a citizen science framework
Source: PLoS One. 2018 Dec 6;13(12):e0207219. doi: 10.1371/journal.pone.0207219 (PMC6283465; doi:10.1371/journal.pone.0207219)
Supplement: S3 Table — Satisfaction of participants in Mr.Banks (n = 234), Dr.Brain (n = 524) and The Climate Game (n = 420). (PDF) [file pone.0207219.s006.pdf]

Table S3: Satisfaction. Satisfaction of participants in Mr.Banks (n=234), Dr.Brain (n=524) and The Climate Game (n=420).

	**Very Positive**	**Positive**	**Neutral**	**Negative**	**Very Negative**
Mr.Banks	-	125	91	18	-
Dr.Brain	245	217	-	49	13
The Climate Change	204	184	-	25	7
