# Supplementary material for: Citizen Social Lab: A digital platform for human behavior experimentation within a citizen science framework
Source: PLoS One. 2018 Dec 6;13(12):e0207219. doi: 10.1371/journal.pone.0207219 (PMC6283465; doi:10.1371/journal.pone.0207219)
Supplement: S1 File — Supplementary notes, figures and tables. (PDF) [file pone.0207219.s007.pdf]

Supplementary Information

Citizen Social Lab: A digital platform for human behaviour experimentation within a citizen science framework.

Julián Vicens,[1,2,3] Josep Perelló[2,3] Jordi Duch,[1,⋆]

[1]Departament d'Enginyeria Informàtica i Matemàtiques
Universitat Rovira i Virgili, 43007, Tarragona, Spain
[2]Departament de Física de la Matèria Condensada
Universitat de Barcelona, 08028 Barcelona, Spain
[3]Universitat de Barcelona Institute of Complex Systems UBICS
Universitat de Barcelona, 08028 Barcelona, Spain

Contents

1 Supplementary Notes **3**

1.1 Platform screenshots 3

1.2 Questionnaries 4

1 Supplementary Notes

1.1 Platform screenshots

In this section we present some screenshots of the different experiments that have been deployed within the platform. First, in Fig S1 we present the initial screenshots of three of the experiments, Mr. Banks, Dr. Brain and The Climate Game. In these screens we observe the characters designed as part of the experimental setup to create a narrative and attract the attention of the public to the experiment. In Fig S2 we show the user interface of these three experiments. In all the cases the interface uses a simplistic but visually appealing approach to present the dilemmas to the participants. Additionally, in all the experiments there is a tutorial where the participants learn the mechanics of the game and the user interface, we can see some of the screens of the tutorial of The Climate Game experiment in Fig S3.

1.2 Questionnaries

After the experiments, as a last stage of it, for each group of subjects we asked a questionnaire with a set of questions focused on the experimental framing, decision-making process and user experience. The questionnaire was also presented identically in three languages (Catalan, Spanish and English), here we present the questions in English used in this particular paper.

Mr.Banks

Question 1: *What is your general opinion about this experience?* Answers: a.*Positive*; b.*Regular*; c.*Negative.*

Dr.Brain

Question 11: *Rate your experience.* Answers: a.*Very much*; b.*Somewhat*; c.*Not really*; d.*Not at all.*

The climate game

Question 2: *Did you like the experience?* Answers: a.*Very much*; b.*Somewhat*; c.*Not really*; d.*Not at all.*
